# Management and outcomes of acute appendicitis in children during the COVID-19 pandemic: a systematic review and meta-analysis

**DOI:** 10.1007/s00383-023-05594-9

**Published:** 2023-11-28

**Authors:** Maria Enrica Miscia, Giuseppe Lauriti, Dacia Di Renzo, Valentina Cascini, Gabriele Lisi

**Affiliations:** 1 Department of Medicine and Aging Science, “G. d’Annunzio” University of Chieti-Pescara, Via L. Polacchi 11, 66100 Chieti, Italy; 2 Pediatric Surgery Unit, Spirito Santo” Hospital of Pescara, Pescara, Italy

**Keywords:** Acute appendicitis, COVID-19, Children, Systematic review, Meta-analysis

## Abstract

**Abstract:**

The COVID-19 pandemic has changed the way to manage the emergencies, as people faced fear of the hospitals, with possible delay in the diagnosis. Moreover, clinicians had to rearrange protocols for diagnosis and treatment. We aimed to assess whether COVID-19 pandemic influenced severity of inflammation, management, and outcomes of acute appendicitis (AA), when compared to the pre-COVID era. Using defined search strategy, two independent investigators identified those studies comparing pediatric AA during COVID-19 pandemic *versus* the pre-COVID-19 period. Meta-analysis was performed using RevMan 5.3. Data are mean ± SD. Of 528 abstracts, 36 comparative studies were included (32,704pts). Time from symptoms onset to surgery was longer during the pandemics compared to the pre-COVID-19 (1.6 ± 0.9 *versus* 1.4 ± 0.9 days; p < 0.00001). Minimally Invasive Surgery was similar during COVID-19 (70.4 ± 30.2%) *versus* control period (69.6 ± 25.3%; p = ns). Complicated appendicitis was increased during the pandemics (35.9 ± 14.8%) compared to control period (33.4 ± 17.2%; p < 0.0001). Post-operative complications were comparable between these two groups (7.7 ± 6.5% *versus* 9.1 ± 5.3%; p = ns). It seems that the COVID-19 pandemic influenced the time of diagnosis, severity of inflammation, and type of surgery. However, the number of post-operative complications was not different between the two groups, leading to the conclusion that the patients were correctly managed.

**Level of Evidence:**

Level 3 Meta-analysis on Level 3 studies

**Supplementary Information:**

The online version contains supplementary material available at 10.1007/s00383-023-05594-9.

## Introduction

Since the breakthrough of the Coronavirus-19 (COVID-19) pandemic, there have been a change and a rearrangement both in the society and in the worldwide healthcare [1–3].

During the lockdown, people were less prone to attend the emergency department (ED) due to the fear of contracting the COVID-19, leading to a delayed diagnosis of several diseases [1, 3–7].

Acute appendicitis (AA) is the most common pediatric surgical emergency, and its severity is strictly related to the time of diagnosis: a delayed diagnosis increases the risk of developing complications, such as abscess, peritonitis, sepsis, and wound infection [5, 8].

The gold standard of care for acute appendicitis is appendectomy (through a minimally invasive or open approach). Even if the non-operative management is a well-established procedure in adults, its use among children is not completely defined up to now [2, 8–10].

Although the COVID-19 affects adults more than children, the pandemic has also influenced the management of the pediatric surgical patient [2, 3, 11–13]. Coronavirus infection, in fact, can present with gastrointestinal symptoms in both adults and children, thus increasing the risk of misdiagnoses [2, 10, 14]. Moreover, during the lockdown, the surgical activity has been reduced to the sole emergency surgery and the conversion of peripheral hospitals into COVID hospitals has increased the risk of delayed diagnosis of acute appendicitis [2, 8, 11, 13].

The aim of our study was to assess whether the COVID-19 pandemic influenced the management of AA in children in:

- Diagnosis (age at diagnosis, time from symptoms onset and hospital presentation)
- Severity of inflammation
- Management (non-operative management, minimally invasive surgery and/or open surgery)
- Outcomes (length of hospital stay and post-operative complications).

## Material and methods

### Data sources and study selection

This study was registered on the international prospective register of systematic reviews PROSPERO (registration #CRD42022325941) (National institute for Health Research) [15]. The systematic review was drafted according to the Preferred Reporting Items for Systematic Reviews and Meta-Analyses (PRISMA) statement [16].

**Fig. 1:**
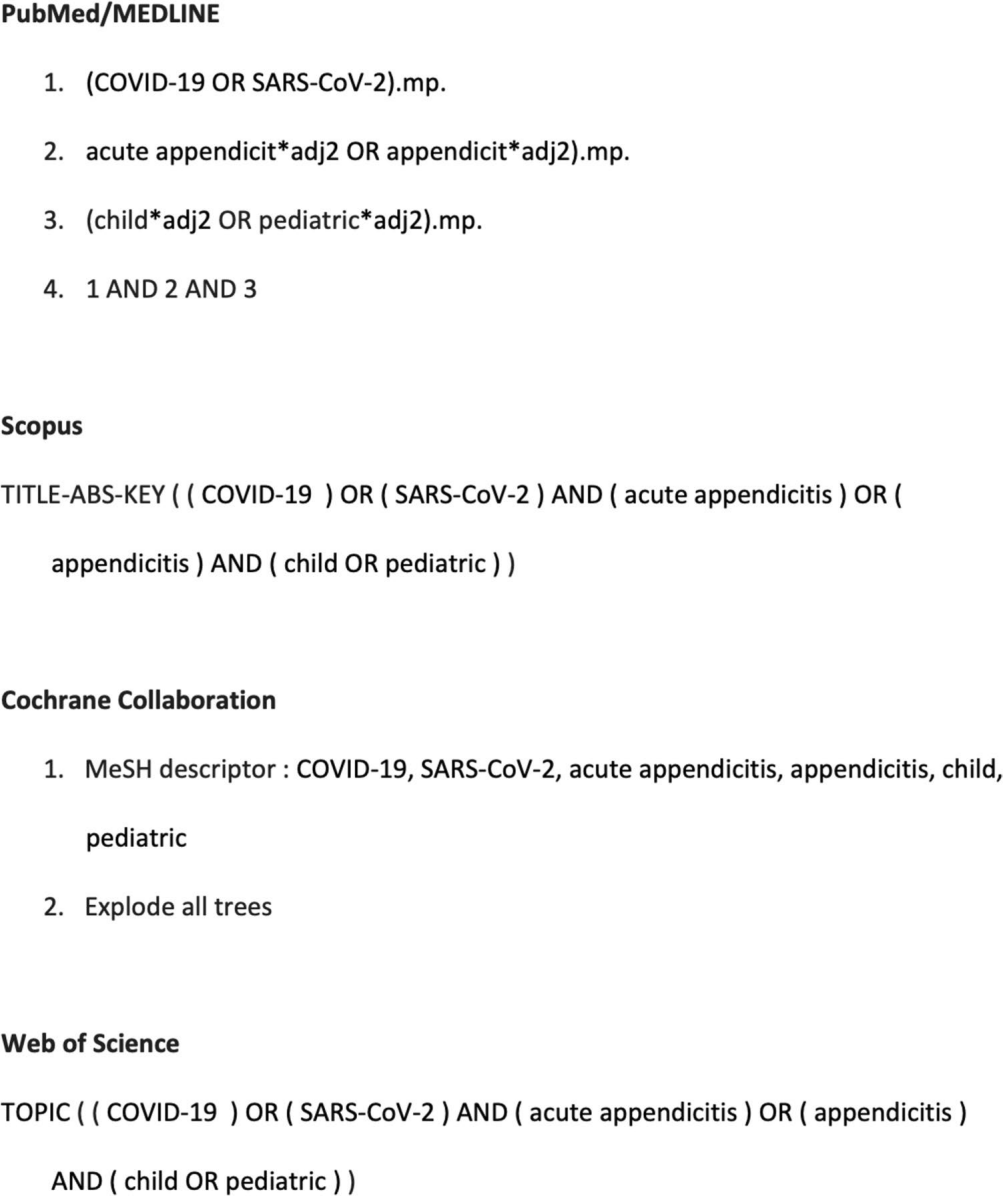
Search strategy

A systematic review of the English literature was made using a defined search strategy (Table 1). Two investigators (MEM, GLa) independently searched scientific databases (PubMed, Cochrane Collaboration, Scopus, and Web of Science) looking for studies reporting on acute appendicitis during the COVID19 pandemic in children published up to September 2023. MeSH headings and terms used are “Acute appendicitis”, “Appendicitis”, “COVID-19”, “SARS-CoV-2”, ‘‘Pediatric”, and “Children” (Fig. 1). Reference lists were searched to identify relevant cross-references. Case reports, opinion articles, experimental studies, and case series with less than 10 patients were excluded. All grey literature publications (i.e. reports, theses, conference proceedings, bibliographies, commercial documentations, and official documents not published commercially) were excluded. Full text articles of potentially eligible studies were retrieved and independently assessed for suitability by two investigators (MEM, GLa). We included all studies (trials, cohort, and case–control) that reported at least one outcome of interest. Furthermore, we included in the meta-analysis only those studies comparing the management of acute appendicitis in children before and after the spreading of COVID19 pandemic. If two or more studies had overlapping patient cohorts, for each outcome measure we included only the article with the largest number of patients. Any disagreement over the eligibility of a specific study was resolved through the discussion with a third author (GLi).

**Table 1:** Defined search strategy

Publication	
Language	English
Time period	January 1950–September 2023
Subject	Human studies
Study type	Retrospective
	Prospective
	Case–control
	Cohort
Excluded	Case-report
	Case series (< 10 patients)
	Editorials
	Letters
	Grey literature
Keywords	COVID-19
	SARS-CoV-2
	Acute appendicitis
	Appendicitis
	Children
	Pediatric

### Statistical analysis

Categorical variable frequencies were compared using Pearson’s chi-square test or the two-tailed Fisher exact probability test, as appropriate. When median and range were reported, mean ± SD were estimated, as previously reported [17]. Meta-analysis of comparative studies was conducted with RevMan 5.4 [18]. Data are presented as risk ratio (RR) for categorical variables, and mean differences (MD) for continuous variables, along with 95% confidence intervals (CI) using the random-effects model, with p values shown for Z test for overall significance and I^2^ statistic for heterogeneity. A p-value < 0.05 was considered statistically significant. Data are expressed as mean ± SD.

### Quality assessment

Risk of bias for individual studies was assessed in duplicate (DDR and VC) using the methodological index for non-randomized studies (MINORS) [19]. Differences between the two reviewers (DDR and VC) were resolved through consensus and discussion with a third author (GLa). The total score for this 12-item instrument ranges 0–24 points with a validated “gold standard” cut-off of 19.8. We assessed the methodological quality for each outcome by grading the quality of evidence using the Grading of Recommendations Assessment, Development and Evaluation (GRADE) methodology [20]. Quality of evidence was rated as high, moderate, low, and very low for each outcome. Observational studies start with a low quality of evidence. The quality of evidence was rated down in the presence of risk of bias, inconsistency, indirectness, imprecision, and publication bias. For assessment of risk of bias in observational studies, we used the MINORS instrument. Inconsistency was determined according to heterogeneity. We produced I^2^ values to assess heterogeneity. I^2^ value of 0–40, 30–60, 50–90, and 75–100% were considered as low, moderate, substantial, and considerable heterogeneity, respectively. Imprecision was assessed using optimal information size (OIS), which was based on 25% relative risk reduction, 0.05 of α error and 0.20 of β error [21].

## Results

**Fig. 2:**
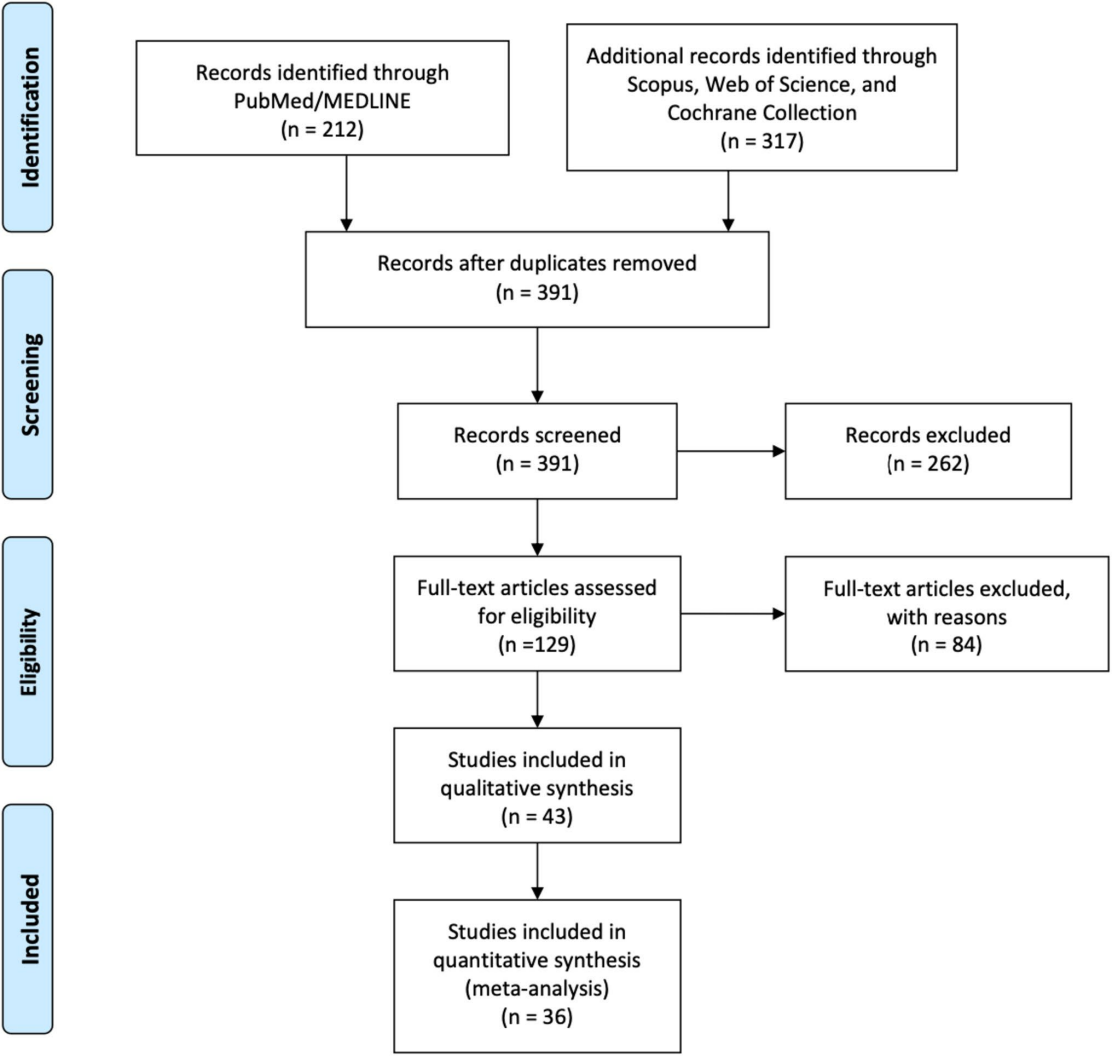
Diagram of workflow in the systematic review and meta-analysis

Of 528 title/abstract screened, 129 full-text articles were analyzed, 43 studies entered the qualitative analysis [1–11, 13, 14, 22–51], and 36 papers were included in the meta-analysis [1–8, 11, 13, 22, 23, 25–28, 30–44, 46–50] (32,704 pts, Fig. 2).

**Fig. 3:**
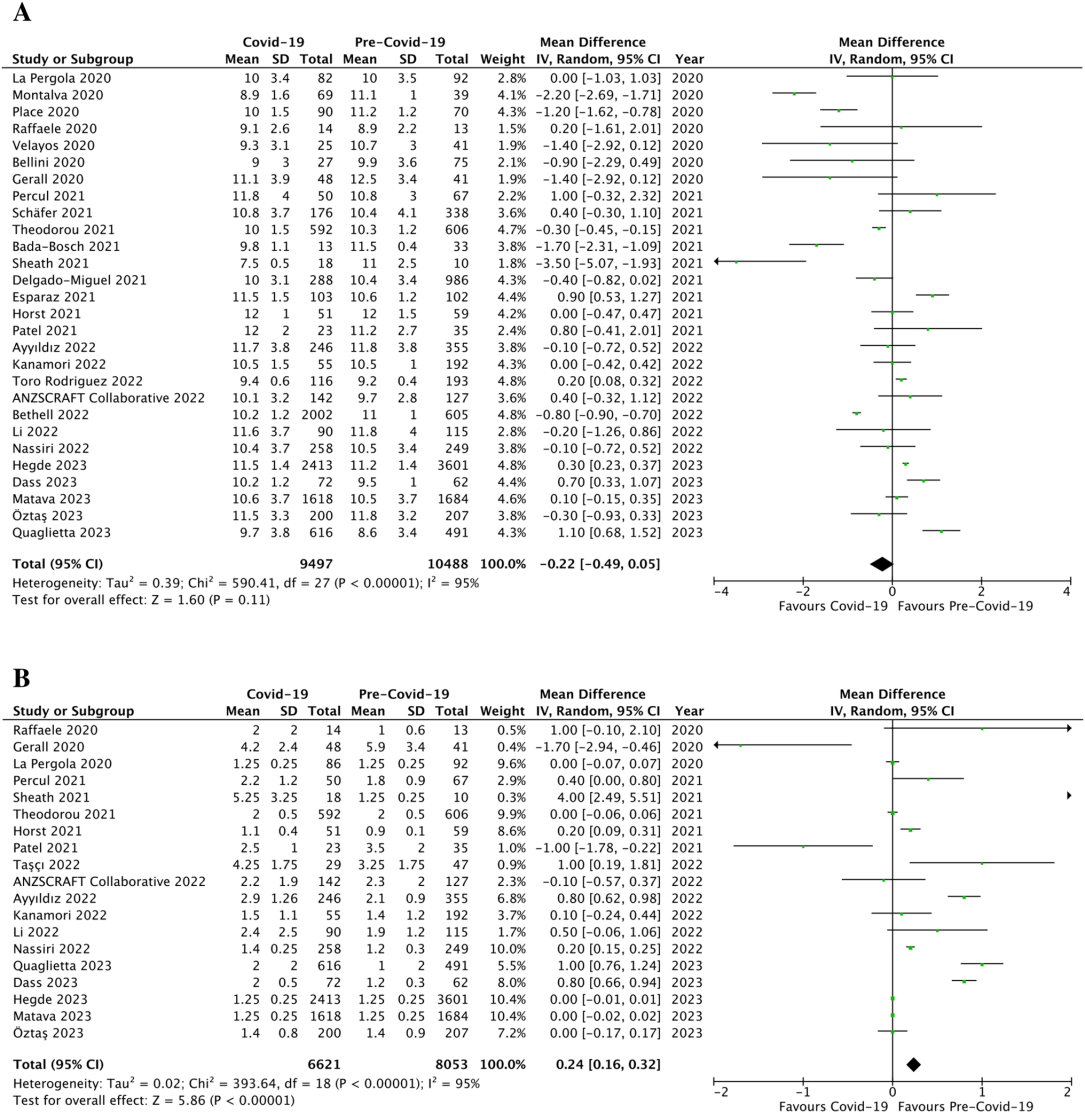
Pre-operative data: forest plot comparison of patients during Covid-19 pandemic *versus* pre-Covid-19 era with regards age at presentation (**a**) and time from symptoms onset to surgery (**b**)

The age at presentation of symptoms was similar during COVID-19 pandemic (10.6 ± 1.2 years) when compared to the pre-COVID-19 era (10.7 ± 1.2 years; p = ns, MD -0.22, 95% confidence intervals (CI) [-0.49, 0.05], I^2^ = 95%; Fig. 3a). The mean time from symptoms onset to surgery was significantly lengthened in the COVID-19 period compared to the pre-pandemic era (1.6 ± 0.9 *versus* 1.4 ± 0.9 days, respectively; p < 0.00001, MD 0.24, 95% CI [0.16, 0.32], I^2^ = 95%; Fig. 3b).

**Fig. 4:**
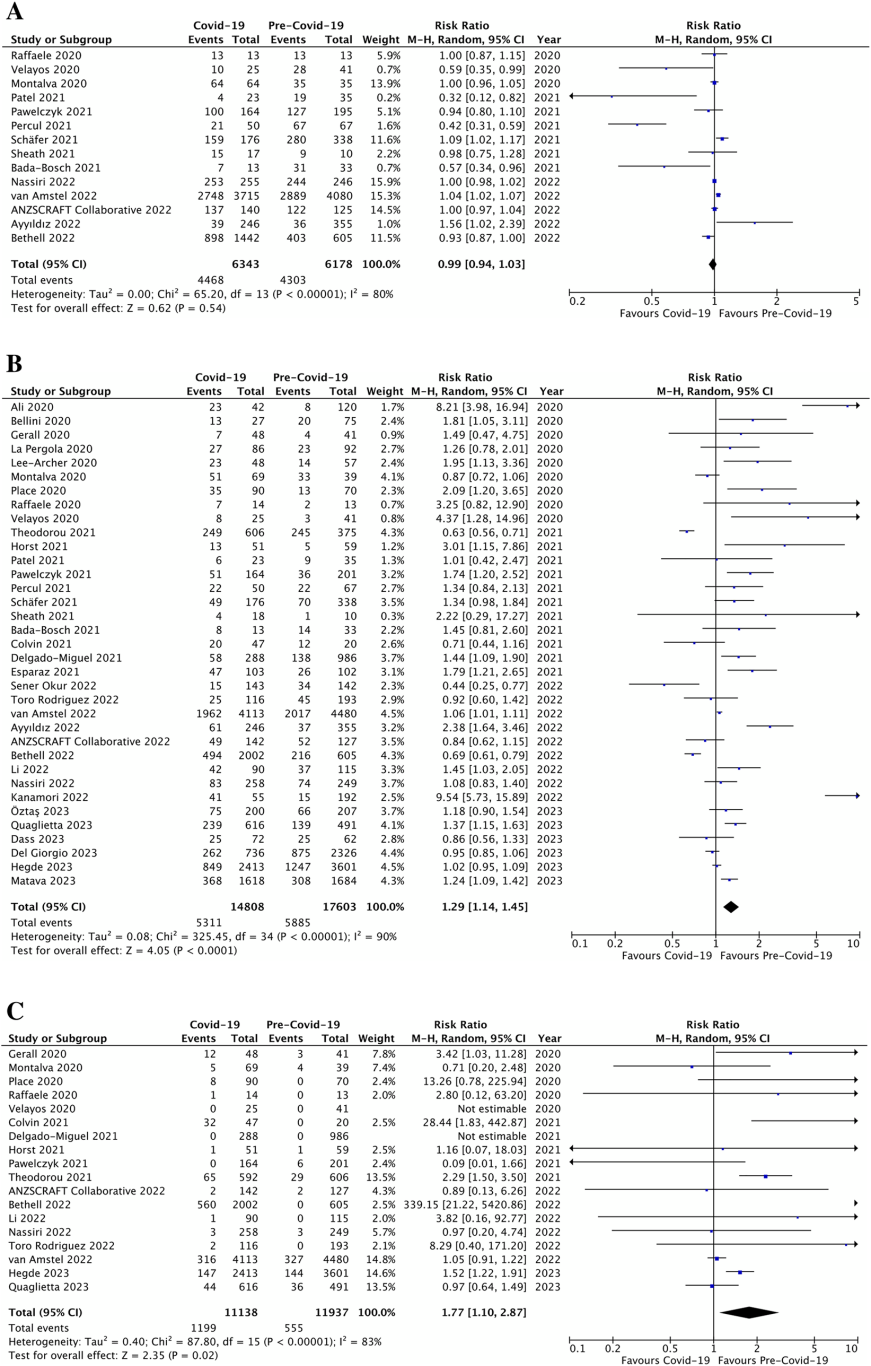

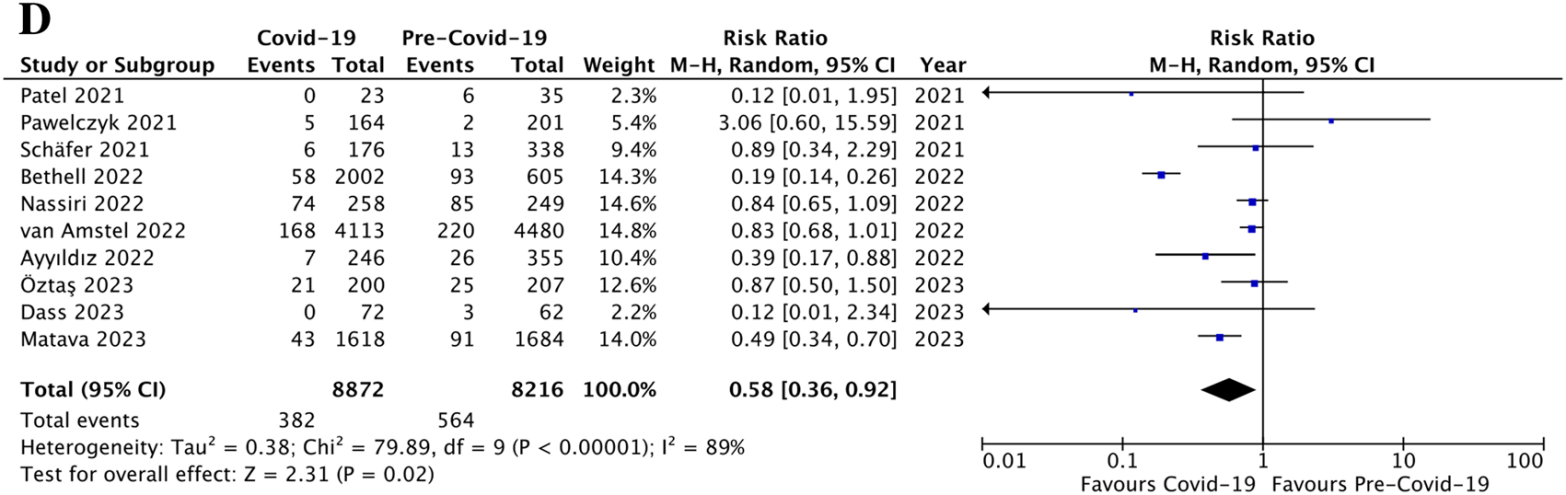
Management of AA: forest plot comparison of patients during Covid-19 pandemic *versus* pre-Covid-19 era with regards minimally invasive appendectomies (**a**), the incidence of complicated appendicitis (**b**), non-operative management of acute appendicitis (**c**), and the rate of negative appendicitis (**d**)

When reported, minimally invasive surgery (MIS) did not appear to be decreased during the pandemic (4,468/6,343 cases, 70.4 ± 30.2%) *versus* the control period (4,303/6,178 cases, 69.6 ± 25.3%; p = ns, RR 0.99, 95% CI [0.94, 1.03], I^2^ = 80%, Fig. 4a). However, the number of complicated appendicitis was significantly increased during the pandemic (5,311/14,808 children, 35.9 ± 14.8%) compared to the control period (5,885/17,603 children, 33.4 ± 17.2%; p < 0.0001, RR 1.29, 95% CI [1.14, 1.45], I^2^ = 90%; Fig. 4b). Also, the non-operative management (NOM) was significantly increased during the COVID-19 pandemic compared to the previous period (1,199/11,138 patients, 10.8 ± 16.5% *versus* 555/11,937 patients, 4.6 ± 3.3%, respectively; p = 0.02; RR 1.77, 95% CI [1.10, 2.87], I^2^ = 83%; Fig. 4c). Moreover, the incidence of negative appendicitis was significantly decreased during the pandemic (382/8,872 children, 4.3 ± 8.5%) compared to the control period (564/8,216 children, 6.9 ± 9.8%); p = 0.02, RR 0.58, 95% CI [0.36, 0.92], I^2^ = 89%; Fig. 4d).

**Fig. 5:**
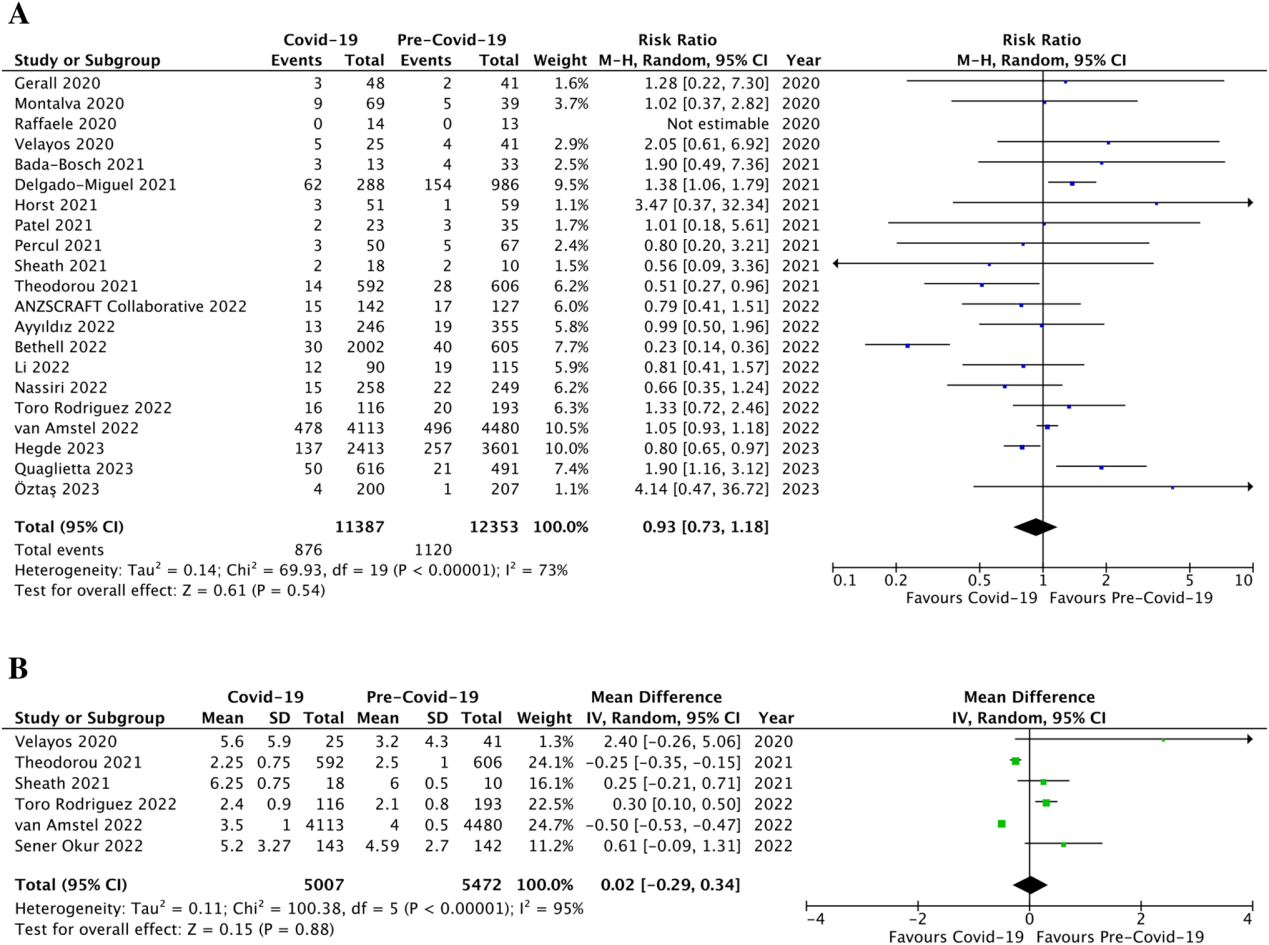
Post-operative outcomes: forest plot comparison of patients during Covid-19 pandemic *versus* pre-Covid-19 era with regards post-operative complications (**a**) and the length of hospital stay (**b**)

Nonetheless, we did not find a statistically significant increase of post-operative complications during the COVID-19 pandemic (876/11,387 patients, 7.7 ± 6.5%) compared to the previous period (1,120/12,353 patients, 9.1 ± 5.3%; p = ns, RR 0.93, 95% CI [0.73, 1.18], I^2^ = 73%, Fig. 5a). Finally, the length of hospital stay (LOS) was similar between the pandemic period (3.4 ± 2.1 days) and the pre-COVID-19 era (3.8 ± 1.4 days; p = ns, MD 0.02, 95% CI [-0.29, 0.34], I^2^ = 95%; Fig. 5b).

## Discussion

Acute appendicitis is the most common pediatric surgical emergency with up to 8% of children complaining of abdominal pain having a diagnosis of appendicitis [8].

The most common complication of AA is perforation, and its incidence is directly correlated with the duration of symptoms and age of the patient [5]. The higher rate of complicated appendicitis among very young children has to be referred to their difficulty to define their symptoms [5].

The increased rate of complicated appendicitis during the COVID-19 pandemic reported by several Authors was explained by the fear of contracting the virus in the hospitals, leading to a late referral to the emergency department and therefore to a delayed diagnosis [3, 5–8, 10, 12–14]. As a matter of fact, in our study we have found an overall increased time-lapse from symptoms onset to surgery that could be related to the finding of a significantly higher number of complicated appendicitis during the pandemic compared to the pre-pandemic era. Moreover, in our study the mean age at presentation was similar among the two study groups, thus removing any possible bias given by different age populations.

It has also been postulated that the COVID-19 infection itself could increase the rate of complicated appendicitis, due to the gastrointestinal manifestation of the disease and this assumption may also explain our results [6].

The management of children with AA during the pandemic is also controversial. Some Authors, in fact, postulated an increased risk of contagion during laparoscopy due to aerosolization of peritoneal fluids [2, 10, 22]. Therefore, the ESPES (European Society of Pediatric Endoscopic Surgeons) recommended the use of a closed system for CO_2_ insufflation and de-sufflation, limiting the use of the electrocautery [51]. When analyzing our data, in fact, we did not find a reduced use of the minimally invasive surgery (MIS) during the pandemic when compared to the pre-COVID-19 period. However, we did find fewer negative appendicitis during the pandemic when compared to the pre-COVID-19 period.

Another question raised during the COVID-19 era is the use of the NOM as the first-line treatment of the AA. Some surgeons, in fact, preferred to avoid the use of NOM to reduce the overall length of hospital stay and to avoid the risk of failure of NOM that may have led to an increased risk of complications, length of hospital stays, and readmissions [2, 5, 8, 12]. Some others, however, preferred the use of NOM to limit hospital access and to reduce the number of surgical procedures, and this was especially true during the strict lockdown [9, 52]. Moreover, the ESPES suggested to consider the use of NOM, whenever safe for the patient [51]. Indeed, in our study, we found an overall increased use of the NOM in children during the pandemic when compared to the same period pre-COVID-19.

Nonetheless, despite the higher incidence of complicated AA, we did not find an increased number of postoperative complications as well as a lengthened hospital stay, as demonstrated by other Authors, thus leading to the conclusion that children were appropriately treated [53].

### Limitation of the study

We are aware of the limitations of our study, which rely on the quality of the studies and data available in the literature, as any other meta-analysis.

All the 36 studies included in the meta-analysis were retrospective observational studies [1–8, 11, 13, 22, 23, 25–28, 30–44, 46–50]. None of the papers provided sample size calculations. As expected, a blinded evaluation of objective endpoints was not possible and groups were not contemporary, because of different time-period between cases and controls. Moreover, none of the study have reported with regards to the loss to follow-up and there were a broad lack of data regards the length of follow-up. Therefore, in our meta-analysis, none of the studies reached the gold standard cut-off on MINORS of 19.8 out of 24 (Supplementary file 1).

According to the GRADE methodology, the quality of evidence of the meta-analysis was low with regards all the pre-operative data (age at presentation, time from symptoms onset to surgery), the management of AA (minimally invasive appendectomies, incidence of complicated appendicitis, percentage of NOM, and percentage of negative appendicitis) and post-operative outcomes (incidence of complication) (Table 2). Although the data were obtained from a considerable number of studies, their considerable heterogeneity could generate possible bias.

**Table 2:** GRADE evidence profile [20] for the present meta-analysis

Quality assessment								No. of patients		Effect		Quality
No. of studies	Study design	Risk of bias	Inconsistency	Indirectness	Imprecision	Other conside-rations		Cases	Controls	Relative (95% CI)	Absolute (95% CI)	
Age at presentation during Covid-19 vs. pre-Covid-19 era								Covid-19	Pre-Covid-19			
28	OS	Moderate^a^	Considerable	Not serious	Serious^b^	None		9,474	10,488	–	MD 0.22 lower (from 0.49 lower to 0.05 higher)	⊗ ⊗ OO Low
Time from symptoms onset to surgery during Covid-19 vs. pre-Covid-19 era								Covid-19	Pre-Covid-19			
19	OS	Moderate^a^	Considerable	Not serious	Serious^b^	None		6,621	8,053	–	MD 0.24 higher (from 0.16 to 0.32 higher)	⊗ ⊗ OO Low
MIS appendectomies during Covid-19 vs. pre-Covid-19 era								Covid-19	Pre-Covid-19			
14	OS	Moderate^a^	Substantial	Not serious	Serious^b^	None		4,468/6,343 (70.4%)	4,303/6,178 (69.6%)	RR 0.99 (0.94, 1.03)	8 more per 1000 (from 48 more to 24 fewer)	⊗ ⊗ OO LOW
Incidence of complicated appendicitis during Covid-19 vs. pre-Covid-19 era								Covid-19	Pre-Covid-19			
35	OS	Moderate^a^	Considerable	Not serious	Serious^b^		None	5,311/14,808 (35.9%)	5,885/17,603 (33.4%)	RR 1.29 (1.14, 1.45)	25 more per 1000 (from 12 to 39 more)	⊗ ⊗ OO Low
NOM during Covid-19 vs. pre-Covid-19 era								Covid-19	Pre-Covid-19			
18	OS	Moderate^a^	Substantial	Not serious	Serious^b^		None	1,199/11,138 (10.8%)	555/11,937 (4.6%)	RR 1.77 (1.10, 2.87)	62 more per 1000 (from 8 to 151 more)	⊗ ⊗ OO LOW
Negative appendicitis during Covid-19 vs. pre-Covid-19 era								Covid-19	Pre-Covid-19			
10	OS	Moderate^a^	Considerable	Not serious	Serious^b^		None	382/8,216 (4.3%)	564/8,216 (6.9%)	RR 0.58 (0.36, 0.92)	26 fewer per 1000 (from 40 to 5 fewer)	⊗ ⊗ OO Low
Complications during Covid-19 vs. pre-Covid-19 era								Covid-19	Pre-Covid-19			
21	OS	Moderate^a^	Substantial	Not serious	Serious^b^		None	876/11,387 (7.7%)	1,120/12,353 (9.1%)	RR 0.93 (0.73, 1.18)	14 fewer per 1000 (from 54 fewer to 36 more)	⊗ ⊗ OO LOW
LOS during Covid-19 vs. pre-Covid-19 era								Covid-19	Pre-Covid-19			
6	OS	Moderate^a^	Considerable	Not serious	Serious^b^		None	5,007	5,472	–	MD 0.02 higher (0.29 lower to 0.34 higher)	⊗ ⊗ ⊗ O Very low

*MIS* minimally invasive surgery, *NOM* non-operative management, *LOS* length of hospital stay

^a^Bias due to possible confounding

^b^OIS not met

GRADE Working Group grades of evidence

High quality: Further research is very unlikely to change our confidence in the estimate of effect

Moderate quality: Further research is likely to have an important impact on our confidence in the estimate of effect and may change the estimate

Low quality: Further research is very likely to have an important impact on our confidence in the estimate of effect and is likely to change the estimate

Very low quality: We are very uncertain about the estimate

However, when independently assessed by two authors (DDR and VC) using A Measurement Tool to Assess Systematic Reviews (AMSTAR) [54], the present systematic review and meta-analysis received a decent score (Supplementary file 2).

The PRISMA checklist was then completed (Supplementary file 3).

## Conclusions

The correct management of children with acute appendicitis during the COVID-19 pandemic is still debated.

The number of complicated appendicitis has increased during this period, and it seems to be directly related to the delayed referral to the hospital.

Up to now the use of laparoscopy is not contraindicated in the COVID-positive patients. As a matter of fact, the use of the MIS during the pandemic was not decreased when compared to the pre-pandemic era.

Even if the delayed diagnosis could influence the outcomes, the incidence of complications seems not to be increased during pandemic, thus leading to the conclusion that the choice of the surgical management (either open, MIS, or NOM) was still correct for each patient.

## Supplementary Information

Below is the link to the electronic supplementary material.

Supplementary file1 (DOCX 42 KB)

Supplementary file2 (DOC 36 KB)

Supplementary file3 (DOC 60 KB)

## Data Availability

All data supporting the findings of this systematic review and meta-analysis are available within the paper and its Supplementary files. Further data with regards results (e.g. list of excluded studies with reasons) are available from the corresponding author upon reasonable request.

## Abbreviations

AA: Acute appendicitis
MIS: Minimally invasive surgery
NOM: Non-operative management
