# Supplementary material for: Management and outcomes of acute appendicitis in children during the COVID-19 pandemic: a systematic review and meta-analysis
Source: Pediatr Surg Int. 2023 Nov 28;40(1):11. doi: 10.1007/s00383-023-05594-9 (PMC10684649; doi:10.1007/s00383-023-05594-9)
Supplement: Supplementary file 1 — Supplementary file1 (DOCX 42 KB) [file 383_2023_5594_MOESM1_ESM.docx]

**Supplementary file 1.** Risk of bias assessment for individual studies using methodological index for nonrandomized studies (MINORS) [19].

| **Item** | **Ali [1]** | **Montalva [2]** | **Bellini [3]** | **Moratilla Lapeña [4]** | **Gerall [5]** | **Lee-Archer [6]** | **Place [7]** | **Velayos [8]** | **La Pergola [11]** |
| --- | --- | --- | --- | --- | --- | --- | --- | --- | --- |
| **1. A clearly stated aim** | 2 | 2 | 2 | 2 | 2 | 2 | 2 | 2 | 2 |
| **2. Inclusion of consecutive patients** | 2 | 2 | 2 | 2 | 2 | 2 | 2 | 2 | 2 |
| **3. Prospective collection of data** | 0 | 0 | 0 | 0 | 0 | 0 | 0 | 0 | 0 |
| **4. Endpoints appropriate to the aim of the study** | 2 | 2 | 2 | 2 | 2 | 2 | 2 | 2 | 2 |
| **5. Unbiased assessment of the study endpoint** | 0 | 0 | 0 | 0 | 0 | 0 | 0 | 0 | 0 |
| **6. Follow-up period appropriate to the aim of the study** | 1 | 1 | 1 | 1 | 1 | 1 | 1 | 1 | 1 |
| **7. Loss to follow-up less than 5%** | 0 | 0 | 0 | 0 | 0 | 0 | 0 | 0 | 0 |
| **8. Prospective calculation of the study size** | 0 | 0 | 0 | 0 | 0 | 0 | 0 | 0 | 0 |
| **9. An adequate control group** | 2 | 2 | 2 | 2 | 2 | 2 | 2 | 2 | 2 |
| **10. Contemporary groups** | 1 | 1 | 1 | 1 | 1 | 1 | 1 | 1 | 1 |
| **11. Baseline equivalence of groups** | 0 | 1 | 2 | 2 | 2 | 2 | 2 | 2 | 2 |
| **12. Adequate statistical analyses** | 2 | 2 | 2 | 2 | 2 | 2 | 2 | 2 | 2 |
| **Total score** | **12** | **13** | **14** | **14** | **14** | **14** | **14** | **14** | **14** |

**0** = not reported; **1** = reported but inadequate; **2** = reported and adequate.

Validated “gold standard” cut-off: 19.8.

| **Item** | **Raffaele [13]** | **Bada-Bosch [22]** | **Patel [23]** | **Horst [25]** | **Esparaz [26]** | **Percul [27]** | **Colvin [28]** | **Theodorou [30]** | **Schäfer [31]** |
| --- | --- | --- | --- | --- | --- | --- | --- | --- | --- |
| **1. A clearly stated aim** | 1 | 2 | 2 | 2 | 2 | 2 | 2 | 2 | 2 |
| **2. Inclusion of consecutive patients** | 2 | 2 | 2 | 2 | 2 | 2 | 2 | 2 | 2 |
| **3. Prospective collection of data** | 0 | 0 | 0 | 0 | 0 | 0 | 0 | 0 | 0 |
| **4. Endpoints appropriate to the aim of the study** | 2 | 2 | 2 | 2 | 2 | 2 | 2 | 2 | 2 |
| **5. Unbiased assessment of the study endpoint** | 0 | 0 | 0 | 0 | 0 | 0 | 0 | 0 | 0 |
| **6. Follow-up period appropriate to the aim of the study** | 1 | 1 | 1 | 1 | 1 | 1 | 1 | 2 | 1 |
| **7. Loss to follow-up less than 5%** | 0 | 0 | 0 | 0 | 0 | 0 | 0 | 0 | 0 |
| **8. Prospective calculation of the study size** | 0 | 0 | 0 | 0 | 0 | 0 | 0 | 0 | 0 |
| **9. An adequate control group** | 1 | 2 | 2 | 2 | 2 | 2 | 2 | 2 | 2 |
| **10. Contemporary groups** | 1 | 1 | 1 | 1 | 1 | 1 | 1 | 1 | 1 |
| **11. Baseline equivalence of groups** | 2 | 2 | 2 | 2 | 1 | 2 | 2 | 2 | 2 |
| **12. Adequate statistical analyses** | 2 | 2 | 2 | 2 | 2 | 2 | 2 | 2 | 2 |
| **Total score** | **12** | **14** | **14** | **14** | **13** | **14** | **14** | **15** | **14** |

**0** = not reported; **1** = reported but inadequate; **2** = reported and adequate.

Validated “gold standard” cut-off: 19.8.

| **Item** | **Kanamori [32]** | **Bethell [33]** | **Nassiri [34]** | **ANZSCRAFT Collaborative [35]** | **Li [36]** | **Delgado-Miguel [37]** | **Pawelczyk [38]** | **van Amstel [39]** | **Hedge [40]** |
| --- | --- | --- | --- | --- | --- | --- | --- | --- | --- |
| **1. A clearly stated aim** | 2 | 2 | 2 | 2 | 2 | 2 | 2 | 2 | 2 |
| **2. Inclusion of consecutive patients** | 2 | 2 | 2 | 2 | 2 | 2 | 2 | 2 | 2 |
| **3. Prospective collection of data** | 0 | 0 | 0 | 0 | 0 | 0 | 0 | 0 | 0 |
| **4. Endpoints appropriate to the aim of the study** | 2 | 2 | 2 | 2 | 2 | 2 | 2 | 2 | 2 |
| **5. Unbiased assessment of the study endpoint** | 0 | 0 | 0 | 0 | 0 | 0 | 0 | 0 | 0 |
| **6. Follow-up period appropriate to the aim of the study** | 1 | 1 | 1 | 1 | 1 | 1 | 1 | 1 | 1 |
| **7. Loss to follow-up less than 5%** | 0 | 0 | 0 | 0 | 0 | 0 | 0 | 2 | 0 |
| **8. Prospective calculation of the study size** | 0 | 0 | 0 | 0 | 0 | 0 | 0 | 0 | 0 |
| **9. An adequate control group** | 2 | 2 | 2 | 2 | 2 | 2 | 2 | 2 | 2 |
| **10. Contemporary groups** | 1 | 1 | 1 | 1 | 1 | 1 | 1 | 1 | 1 |
| **11. Baseline equivalence of groups** | 2 | 1 | 2 | 2 | 2 | 2 | 1 | 2 | 2 |
| **12. Adequate statistical analyses** | 2 | 2 | 2 | 2 | 2 | 2 | 2 | 2 | 2 |
| **Total score** | **14** | **13** | **14** | **14** | **14** | **14** | **13** | **16** | **14** |

**0** = not reported; **1** = reported but inadequate; **2** = reported and adequate.

Validated “gold standard” cut-off: 19.8.

| **Item** | **Taşçı [41]** | **Toro Rodríguez [42]** | **Sener Okur [43]** | **Ayyıldız [44]** | **Del Giorgio [46]** | **Quaglietta [47]** | **Öztaş [48]** | **Dass [49]** | **Matava [50]** |
| --- | --- | --- | --- | --- | --- | --- | --- | --- | --- |
| **1. A clearly stated aim** | 2 | 2 | 2 | 2 | 2 | 2 | 2 | 2 | 2 |
| **2. Inclusion of consecutive patients** | 2 | 2 | 2 | 2 | 2 | 2 | 2 | 2 | 2 |
| **3. Prospective collection of data** | 0 | 0 | 0 | 0 | 0 | 0 | 0 | 0 | 0 |
| **4. Endpoints appropriate to the aim of the study** | 2 | 2 | 2 | 2 | 2 | 2 | 2 | 2 | 2 |
| **5. Unbiased assessment of the study endpoint** | 0 | 0 | 0 | 0 | 0 | 0 | 0 | 0 | 0 |
| **6. Follow-up period appropriate to the aim of the study** | 1 | 1 | 1 | 1 | 1 | 1 | 1 | 1 | 1 |
| **7. Loss to follow-up less than 5%** | 0 | 0 | 0 | 0 | 0 | 0 | 0 | 0 | 0 |
| **8. Prospective calculation of the study size** | 0 | 0 | 0 | 0 | 0 | 0 | 0 | 0 | 0 |
| **9. An adequate control group** | 2 | 2 | 2 | 2 | 2 | 2 | 2 | 2 | 2 |
| **10. Contemporary groups** | 1 | 1 | 1 | 1 | 1 | 1 | 1 | 1 | 1 |
| **11. Baseline equivalence of groups** | 2 | 2 | 1 | 2 | 2 | 1 | 2 | 2 | 1 |
| **12. Adequate statistical analyses** | 2 | 2 | 2 | 2 | 2 | 2 | 2 | 2 | 2 |
| **Total score** | **14** | **14** | **13** | **14** | **14** | **13** | **14** | **14** | **13** |

**0** = not reported; **1** = reported but inadequate; **2** = reported and adequate.

Validated “gold standard” cut-off: 19.8.
