# Supplementary material for: Management and outcomes of acute appendicitis in children during the COVID-19 pandemic: a systematic review and meta-analysis
Source: Pediatr Surg Int. 2023 Nov 28;40(1):11. doi: 10.1007/s00383-023-05594-9 (PMC10684649; doi:10.1007/s00383-023-05594-9)
Supplement: Supplementary file 2 — Supplementary file2 (DOC 36 KB) [file 383_2023_5594_MOESM2_ESM.doc]

**Supplementary file 2: AMSTAR criteria [54] for the present systematic reviews and meta-analysis assessed by two authors.**

| Item | DDR | VC |
| --- | --- | --- |
| 1. Was an 'a priori' design provided? | 1 | 1 |
| 2. Was there duplicate study selection and data extraction? | 1 | 1 |
| 3. Was a comprehensive literature search performed? | 1 | 1 |
| 4. Was the status of publication (i.e. grey literature) used as an inclusion criterion? | 1 | 1 |
| 5. Was a list of studies (included and excluded) provided? | 0 | 0 |
| 6. Were the characteristics of the included studies provided? | 1 | 1 |
| 7. Was the scientific quality of the included studies assessed and documented? | 1 | 1 |
| 8. Was the quality of the included studies used appropriately in formulating conclusions? | 0 | 1 |
| 9. Were the methods used to combine the findings of studies appropriate? | 1 | 1 |
| 10. Was the likelihood of publication bias assessed? | 1 | 1 |
| 11. Was the conflict of interest included? | 0 | 0 |
| Total | 8/11 | 9/11 |

0 = No, 1 = Yes
